# Decompensated Cirrhosis after Renal Transplantation: A Case Report

**DOI:** 10.1155/2011/862567

**Published:** 2011-08-15

**Authors:** Vinay Patel, Luis Marsano, Mary Eng

**Affiliations:** ^1^Divisions of Gastroenterology, Hepatology, Nutrition, Department of Medicine, University of Louisville, Ambulatory Care Building, 3rd Floor, Louisville, KY 40292, USA; ^2^Division of Transplantation, Department of Surgery, University of Louisville, Ambulatory Care Building, 2nd Floor, Louisville, KY 40292, USA

## Abstract

Patients with end-stage liver disease with renal failure can be considered for simultaneous liver kidney transplantation. There are, however, no clear guidelines as to the management of the well-compensated cirrhotic patient with end-stage renal disease. We present the case of a patient with cirrhosis who decompensated after renal transplantation. With no indication for liver transplantation, can these patients safely undergo renal transplantation?

## 1. Introduction

When patients with well-compensated cirrhosis develop end-stage renal disease (ESRD), it can be a challenge deciding whether these patients should receive a simultaneous liver kidney transplant, renal transplant, or deemed unsuitable for transplantation. At the time our patient presented to the transplant center, there were no guidelines on how to proceed. An older study suggests patients with cirrhosis not undergo renal transplantation due to its high mortality [1]. We present a case of a patient with Laennec's cirrhosis, Child-Pugh classification A, who decompensated shortly after renal transplantation.

## 2. Case Report

A 34-year-old caucasian male with ESRD due to hypertensive nephrosclerosis was referred to the transplant center for evaluation for renal transplantation. His medical history was significant acute alcoholic hepatitis with biopsy-proven cirrhosis. At that time, he presented with encephalopathy and mild ascites. He had been abstinent of alcohol for one year prior to presentation, and his cirrhosis was complicated only by the presence of grade 2 esophageal varices and splenomegaly, without recurrence of encephalopathy or ascites. As the patient was well compensated, Child-Pugh score A5, it was felt he did not require a liver transplantation and could proceed with a renal transplantation.

After two years on the waiting list, the patient underwent a deceased donor renal transplantation. Induction with a single intravenous dose of Alemtuzumab 30 mg intraoperatively was provided and the patients maintained on dual therapy of Tacrolimus (target level 8–10 ng/mL) and Mycophenolate sodium (720 mg twice daily). The immediate postoperative course was unremarkable and the kidney functioned immediately. His serum creatinine (Cr) nadir was 1.3 mg/dL. Two months after transplantation, the patient complained of increasing abdominal girth, and serum Cr was mildly elevated at 1.6 mg/dL. Ultrasound identified significant ascites. Renal allograft biopsy was without pathology.

Two large-volume paracentesis of 4 liters were performed at 3 and 5 months posttransplant and renal function continued to deteriorate, as demonstrated by an elevated serum Cr 3.6 mg/dL. The ascites fluid was studied to determine the cause. Studies on the fluid was significant for nucleated cells 200/mm^3^, 10% segmented neutrophil, total protein 2.06 g/dL, albumin 1.4 g/dL (serum albumin 3.6 g/dL, giving a serum:ascites albumin gradient 2.6), triglyceride 27 mg/dL, Cr 1.7 mg/dL, and urea 56 mg/dL (serum Cr 1.69 mg/dL, blood urea nitrogen 46 mg/dL). A nuclear medicine renal scan showed no evidence of urine ascites, and cystogram was, likewise, negative for urine leak. In summary, nonhepatic causes of ascites were excluded.

Six months posttransplant, the patient was found to have an elevated portal wedge pressure of 14 mm Hg, and a transjugular portosystemic shunt (TIPS) was placed. Following the TIPS procedure, his renal function returned to his nadir of 1.3 mg/dL and he was referred for liver transplantation.

While being evaluated for liver transplantation, he developed persistent fevers and was found to have an enterococcus urinary tract infection, bacteremia, and subsequently endocarditis. Despite antibiotic treatment, the patient quickly deteriorated with acute renal failure, recurrent ascites, and hepatic encephalopathy. He experienced acute respiratory failure followed by cardiac arrest and expired. Postmortem examination confirmed a cirrhotic liver and suggested an aortic valve vegetation, pyelonephritis, and pneumonia.

## 3. Discussion

Our patient with end-stage renal disease was felt to be an appropriate candidate for renal transplantation. With a known history of cirrhosis, there was a concern for the need for liver transplantation. As the patient was well compensated and he was abstinent from alcohol, his cirrhosis was expected to continue slow resolution by hepatic remodeling [2]. Other than the presence of grade 2 esophageal varices and splenomegaly, he had no other stigmata of end-stage liver disease and was determined to have Child-Pugh score A cirrhosis. In addition, his pretransplant hepatic synthetic function was excellent (serum protein 7.1 g/dL, albumin 4.1 g/dL, and international normalized ratio (INR) 1.08). Although his Model for End-Stage Liver Disease (MELD) score was 21, most of its points originated from his renal dysfunction and dialysis requirement (bilirubin 1.2 mg/dL, INR 1.08, creatinine 5.89 mg/dL). It was felt the risk of liver transplantation was higher than the potential benefit in the presence of nonprogressive liver disease and that our patient did not require a liver transplantation and could proceed with a renal transplantation.

Unless hepatocellular carcinoma develops, well-compensated cirrhotic patients do not require liver transplantation. Many compensated patients with Laennecs cirrhosis do well if alcohol abstinence is maintained [3–5]. 

Despite years of stable liver function, he deteriorated after renal transplantation. Cause of his liver decompensation was not determined. There was no documented intraoperative hypotension nor evidence of drug-induced hepatic injury, and his early postoperative course was uneventful. It was not until two months after transplantation that he developed signs of hepatic decompensation. His only symptom was the development of refractory ascites and worsening renal function. Nonhepatic causes of ascites were excluded, and there was no indication of viral hepatitis or resumption of alcohol. This prompted an estimation of the portal wedge pressure, which was consistent with persistent sinusoidal portal hypertension. The evidence that these were cirrhotic ascites was further supported by the absence of ascites recurrence and improved renal function following the TIPS procedure.

Development of infectious complications worsened his multiorgan dysfunction, and the patient expired. Infection is a well-recognized complication of immunosuppression following organ transplantation. The need for multiple invasive procedures (renal biopsy, paracentesis, and TIPS), cirrhosis and renal failure likely place him at increased risk for sepsis. 

Renal failure in the setting of advanced liver disease has been well discussed. However, studies regarding end-stage renal disease and renal transplantation in the well-compensated cirrhotic patient are scarce. Certainly, if a patient has decompensated cirrhosis and renal failure, simultaneous liver and kidney transplantation can be considered.

A previous study reports an increased mortality and poor graft survival for renal transplant recipients who has cirrhosis [1]. Cause of death in this study was predominantly due to liver lesions, not liver decompensation. A study by Ripoll et al. found that compensated cirrhotics with hepatic venous pressure gradient (HVPG) <10 mm Hg rarely decompensate [6]. A consensus conference went further to suggest that compensated cirrhotics with HVPG <10 mm Hg, without other significant comorbidities, would benefit from renal transplantation [7]. Unfortunately, HVPG was not ascertained in our patient prior to listing for renal transplantation. Early hepatology involvement is essential, and referral to a liver transplant center may be indicated. Since review of the recent literature, we have approved two patients with cirrhosis for renal transplantation after confirming HVPG <10 mm Hg.
